# Physical therapy of patients undergoing first-time lumbar discectomy: a survey of current UK practice

**DOI:** 10.1186/s12891-022-05346-1

**Published:** 2022-05-27

**Authors:** Hanan Alsiaf, Terence W. O’Neill, Michael J. Callaghan, Peter C. Goodwin

**Affiliations:** 1 Centre for Epidemiology Versus Arthritis, Faculty of Biology, Medicine, and Health, Manchester Academic Health Science Centre, The University of Manchester, Manchester, UK; 2 Department of Physiotherapy, King Fahad Military Medical Complex – KFMMC, Dhahran, Kingdom of Saudi Arabia; 3 NIHR Manchester Biomedical Research Centre, Manchester University NHS Foundation Trust, Manchester Academic Health Science Centre, Manchester, UK; 4 Department of Rheumatology, Salford Royal, NHS Foundation Trust, Salford, UK; 5 Department of health Professions, Manchester Metropolitan University, Manchester, UK; 6 Medical, Manchester United Ltd, Manchester, UK

**Keywords:** Lumbar discectomy, Rehabilitation, Physical therapy, Preoperative, Survey

## Abstract

**Background:**

The type, timing, and extent of provision of rehabilitation for lumbar discectomy patients in the UK are currently unknown. The aim of this study was to determine the provision and type of rehabilitation for patients undergoing lumbar discectomy in UK neurosurgical centers.

**Method:**

Physical therapists involved in treating lumbar discectomy patients in UK neurosurgery centers were invited to complete an online survey that asked about the type, timing (preop, postop), and rehabilitation content for patients undergoing lumbar discectomy.

**Results:**

Seventeen UK neurosurgery centers completed the survey. Twelve (36%) responded from the 33 centers targeted as well as an additional five private centers. All participating centers provided a rehabilitation service for lumbar discectomy patients. Rehabilitation was provided preoperatively in *n* = 6 (35%) centers, postoperatively as an inpatient in all centers, and postoperatively as an outpatient in *n* = 14 (82%) centers. Factors that influenced the decision to provide rehabilitation included both external and internal or patient-related factors. Preoperative rehabilitation focused mainly on education, whilst postoperative outpatient rehabilitation focused more on exercises. Rehabilitation consistently included mobility, functional task training, and exercise prescription.

**Conclusions:**

Whilst all neurosurgical centers in this survey provided some form of rehabilitation for patients undergoing LD surgery, the approach remains inconsistent. Rehabilitation was delivered most frequently postoperatively, with one in three centers providing it preoperatively. Rehabilitation content also varied depending on when it was provided. Further research is needed to determine the optimum timing, contents, and target of rehabilitation for patients undergoing LD surgery.

**Supplementary Information:**

The online version contains supplementary material available at 10.1186/s12891-022-05346-1.

## Background

Low back pain (LBP) is one of the leading causes of disability globally, affecting up to 80% of the world’s population at some time during their lives [1]. Lumbar disc disease is one of the most frequent causes of LBP. The frequency of MRI has proven disc herniation increases with age and is almost universal after 60 years [2]; however, many people are asymptomatic or have relatively mild symptoms [3, 4]. For most people, symptoms are controlled with analgesia and rehabilitation; however, for those symptoms that persist and remain a management problem, surgical intervention may be indicated. Surgical intervention is chosen where diagnostic imaging finds a consistent lesion with the clinical presentation of leg pain, and the conservative management has not successfully managed the symptoms [5]. In the United Kingdom (UK), National Health Service (NHS) lumbar disc surgery was performed on 8478 patients in 2013/2014 [6]. Among those who undergo surgery, rehabilitation may help patients return to normal function and achieve their recovery goals [7].

There are no current UK guidelines for LD rehabilitation. The American Physical Therapy Association (APTA) recommendations are to provide postsurgical general education, precautions, exercise, and resuming physical activity for patients with LBP following surgery [8]. General exercises are recommended as there is no conclusive evidence that one type of exercise is superior to another [8]. No recommendations were made regarding the timing of rehabilitation, for example, preoperative or postoperative. Systematic reviews have evaluated the effect of preoperative and postoperative rehabilitation for LD; however, there remains a relative paucity of evidence and best practice remains unclear [9, 10].

National and international surveys [11, 12] have shown that rehabilitation for lumbar surgery is delivered postoperatively in most rehabilitation centers. There are potential advantages in providing rehabilitation preoperatively [13–16]. Data from an Australian survey indicates that preoperative rehabilitation occurs in 25 of 64 centers; however, the timing and extent of provision of rehabilitation for LD patients in the UK are currently unknown. The last national survey on the management of people undergoing LD in the UK was conducted in 2007, though, did not include questions about preoperative rehabilitation [11]. Furthermore, it is not clear what the content of current rehabilitation programs includes. The broad aim of this study was to identify current rehabilitation practices for patients undergoing LD in the UK. The specific objectives were, 1) to investigate current UK practice in the timing of delivery of rehabilitation services for patients undergoing LD and; 2) to explore the range and scope of interventions used.

## Methods

### Design

The design was a cross-sectional online survey. The survey was created and distributed using Select Survey tools (www.SelectSurvey.net). Ethical approval was obtained from the University of Manchester (ref. 2019–6603-11,533).

### Participants

The target population was neurosurgery centers treating LD patients in the UK. One survey was expected from each center completed by a senior physical therapist. Thirty-three centers (NHS and private) were identified by the Brain and Spine Foundation listed on the Royal College of Surgeons website.

### Recruitment

Recruitment took place between August 2019 to March 2020. The centers were approached firstly by posting the survey on the iCSP Network; secondly, by contacting centers by phone/email; thirdly, social media (Facebook, Twitter, and WhatsApp). For those who expressed an interest in taking part, a reminder email was sent after 3 weeks if the survey had not been completed.

### Sample size

This study was descriptive, targeting all centers; therefore, no sample size calculation was undertaken.

### Questionnaire development

The survey questionnaire (Additional file 1) was developed following a literature review [11, 12]. It was designed to determine: the number and type of procedures/week, whether rehabilitation was provided for patients undergoing LD surgery and, if so, who made the referral; the timing of delivery (preoperative/postoperative), the range and scope of interventions, and how these were delivered (group/one-to-one), and whether and how education was provided (written/verbal/online).

Questions were multiple choice with free text options (see Appendix 1). The questionnaire included four sections designed to meet the aims: 1) Setting, including center name, the number of procedures performed per week, surgical specialty, information about referral decisions, and whether the service was part of an Integrated Care Pathway (ICP); following that, a filter question asking about the type of rehabilitation provided ensured navigation to the relevant section; 2) preoperative rehabilitation provision; 3) postoperative inpatient rehabilitation provision; 4) postoperative outpatient rehabilitation provision. In addition, a review step through a back button was provided. The questionnaire was piloted by four clinicians and four academics. Feedback was provided on usability and technical functionality of the online survey, time taken to complete, order of questions, terminology, clarity, and the similarity of questions used to ensure suitability for respondents.

### Data analysis

Anonymous data from the online survey was transferred and analyzed in SPSS (version 25; IBM, Armonk, NY). Descriptive statistics were used to characterize the study population and questionnaire responses.

## Results

The results of the study have been reported according to the STrengthening the Reporting of OBservational Studies in Epidemiology checklist (STROBE) [17] and the CHEecklist for Reporting Results of Internet E-Surveys (CHERRIES) recommendation [18].

### Responses

Responses were received from a total of 17 neurosurgery centers in the UK. Twelve (36%) responded from the 33 centers targeted as well as an additional five private centers. The completion rate, i.e., the percentage of users who finished the survey compared to those who started it, was (54%). The geographical distribution of the centers included the East Midlands, Northeast and Yorkshire, Northwest, Southeast, Southwest, and Wales (Table 1). There were no responses from centers in London, Scotland, or Northern Ireland.

**Table 1:** The rehabilitation content according to current guidelines and the geographical location of the centers

Center ID	Region	Preoperative		Postoperative(in)		Postoperative(out)		Reflects APTA guidelines
		Ed	Ex	Ed	Ex	Ed	Ex	
1	Yorkshire and Humber			✓	✓	✓	✓	✓
2	Yorkshire and Humber	✓	✓	✓	✓	✓	✓	✓
3	Northwest	✓	✓	✓	✓	✓	✓	✓
4	Northwest			✓	✓			✓
5	Northwest			✓	✓			✓
6	Northwest					✓	✓	✓
7	Northwest			✓	✓	✓	✓	✓
8	West Midlands	✓	✓	✓	✓	✓	✓	✓
9	West Midlands			✓				
10	East Midlands			✓	✓	✓	✓	✓
11	East Midlands			✓	✓	✓	✓	✓
12	Wales	✓	✓	✓	✓	✓	✓	✓
13	Wales			✓	✓	✓	✓	✓
14	Southeast	✓	✓	✓	✓	✓	✓	✓
15	Southwest			✓	✓	✓	✓	✓
16	southwest			✓	✓	✓	✓	✓
17	Southwest	✓	✓	✓	✓	✓	✓	✓

*Ed* education, *Ex* exercises

### Frequency of procedure

**Fig. 1:**
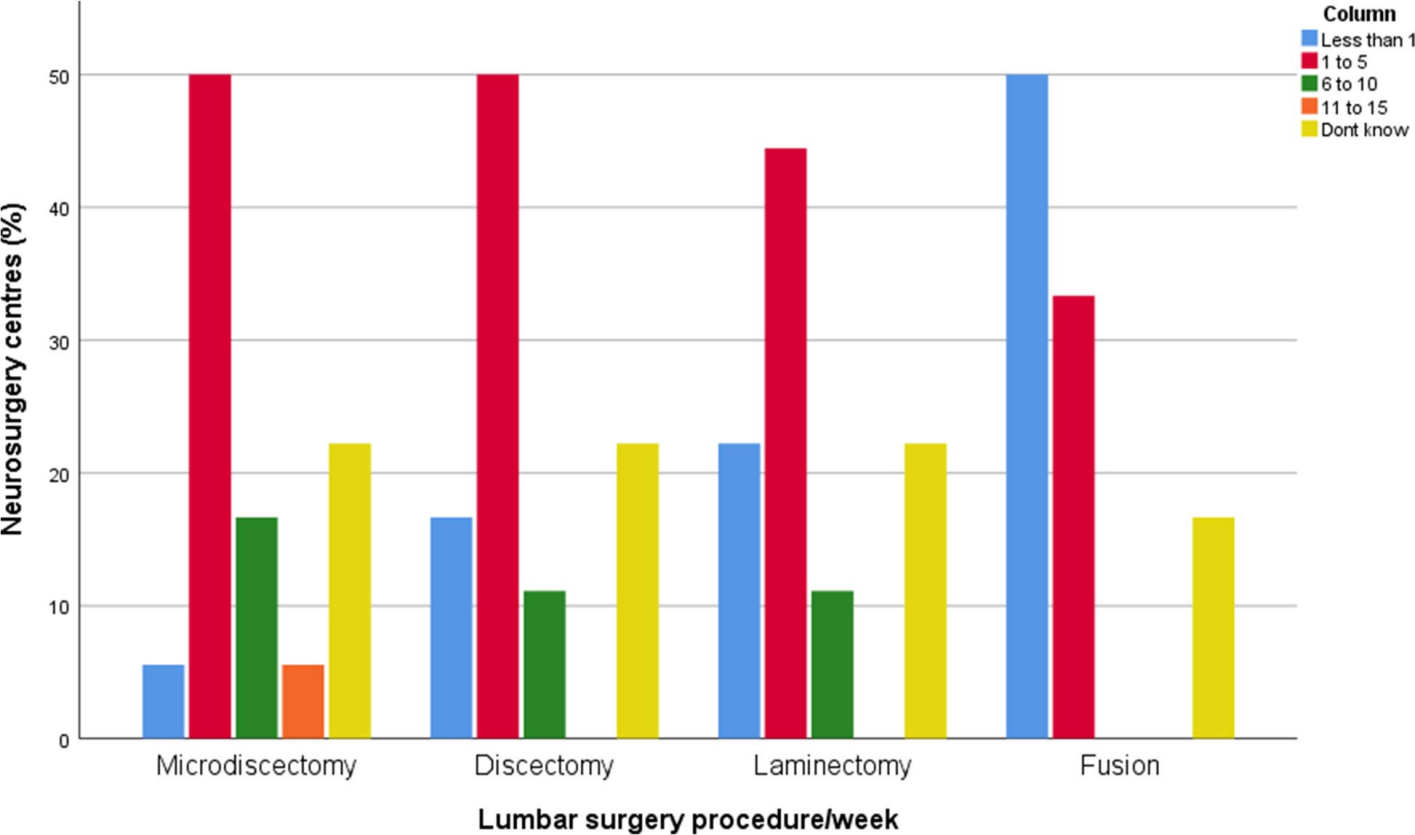
Frequency of Spine Procedures performed per week

Microdiscectomy was reported as the most common procedure which was performed. In the majority of centers, LD, microdiscectomy, and laminectomy surgery were performed on between 1 to 5 patients/week. Lumbar fusion surgery was performed less frequently (Fig. 1).

### Integrated care pathway or healthcare protocol

Responses from seven centers (41%) indicated that they were part of an ICP or healthcare protocol, 8 (47%) were not, and 2 (12%) were unsure.

### Timing of rehabilitation

All seventeen centers provided postoperative inpatient rehabilitation for patients undergoing LD, and 14 (82%) provided postoperative outpatient rehabilitation. Six (35%) reported delivering rehabilitation preoperatively. Eight (47%) provided both postoperative inpatient and outpatient rehabilitation, while 3 (18%) provided only inpatient rehabilitation.

Of the six centers providing preoperative rehabilitation, three centers invited all patients, while the other three invited only some patients. Of the 17 centers providing postoperative inpatient rehabilitation, fifteen offered rehabilitation to all patients, while two invited only a proportion. Of the fourteen centers providing postoperative outpatient rehabilitation, nine invited all patients after discharge, while in five, it was arranged if considered necessary.

Factors influencing when to provide services included external factors, such as therapist experience about the required rehabilitation, surgeon protocols or preferences, or patient related factors such as functional or mobility status. Additional factors influencing the provision of preoperative rehabilitation included time limitations, staffing, and distance for patients to travel. For inpatient postoperative rehabilitation, it included multilevel surgery, and for outpatient postoperative rehabilitation, it included lack of current evidence, preadmission or admission processes, pain intensity, multilevel surgery, and comorbidities.

### Rehabilitation goals

**Fig. 2:**
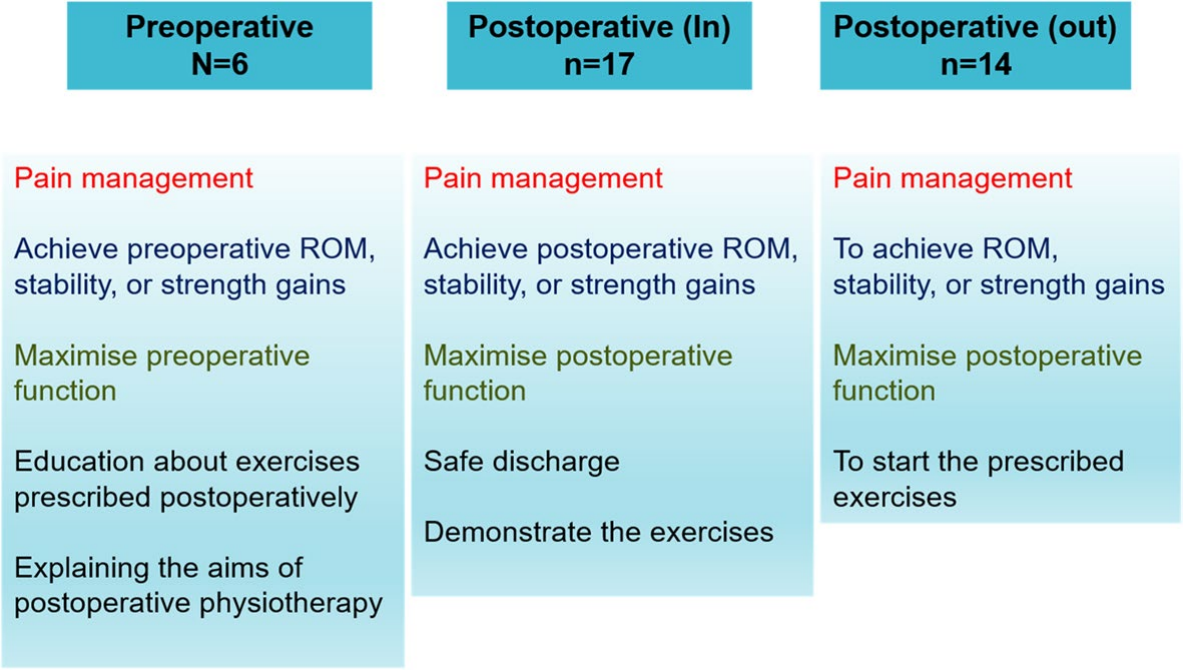
Rehabilitation goals by the timing of therapy

There were differences between preoperative and postoperative inpatient and outpatient rehabilitation goals (Fig. 2). Preoperative rehabilitation focused on education about exercises and induction to postoperative rehabilitation aims. Postoperative inpatient rehabilitation focused on safe discharge and demonstrating exercises. Postoperative outpatient rehabilitation focused on starting the prescribed exercises and maximizing individual postoperative function.

### Timing, frequency, and format of rehabilitation

Of the six centers providing preoperative rehabilitation, those who delivered this prior to hospital admission used a class format (*n* = 2), whereas those who delivered this following admission used a one-to-one format (*n* = 4). All centers offering preoperative rehabilitation provided verbal and written patient education, while two centers provided additional online information.

The majority of centers (*n* = 12) delivered postoperative inpatient rehabilitation the day following surgery, while 5 delivered it on the same day. The frequency of sessions varied between centers from once a day (*n* = 14), twice a day (*n* = 2), and twice if the patient stayed a second night (*n* = 1). Postoperative patient education was provided using verbal and written information in *n* = 13 centers, while n = 4 provided only verbal information.

Postoperative outpatient rehabilitation usually started within 2 weeks following discharge (*n* = 6), 2 to 6 weeks following discharge (*n* = 5), while in 3 centers, rehabilitation was arranged depending on patient needs. Outpatient appointments were provided once a week (*n* = 9), three or more/week (*n* = 2), once every 2 weeks (n = 2), and dependent on patient needs (n = 1). Outpatient services were provided most commonly as one to one (*n* = 11), class (n = 1), or both (n = 2). Outpatients were provided with verbal information (*n* = 12), written information (n = 9), online information (n = 2), and video information (*n* = 1).

### Rehabilitation content

**Fig. 3:**
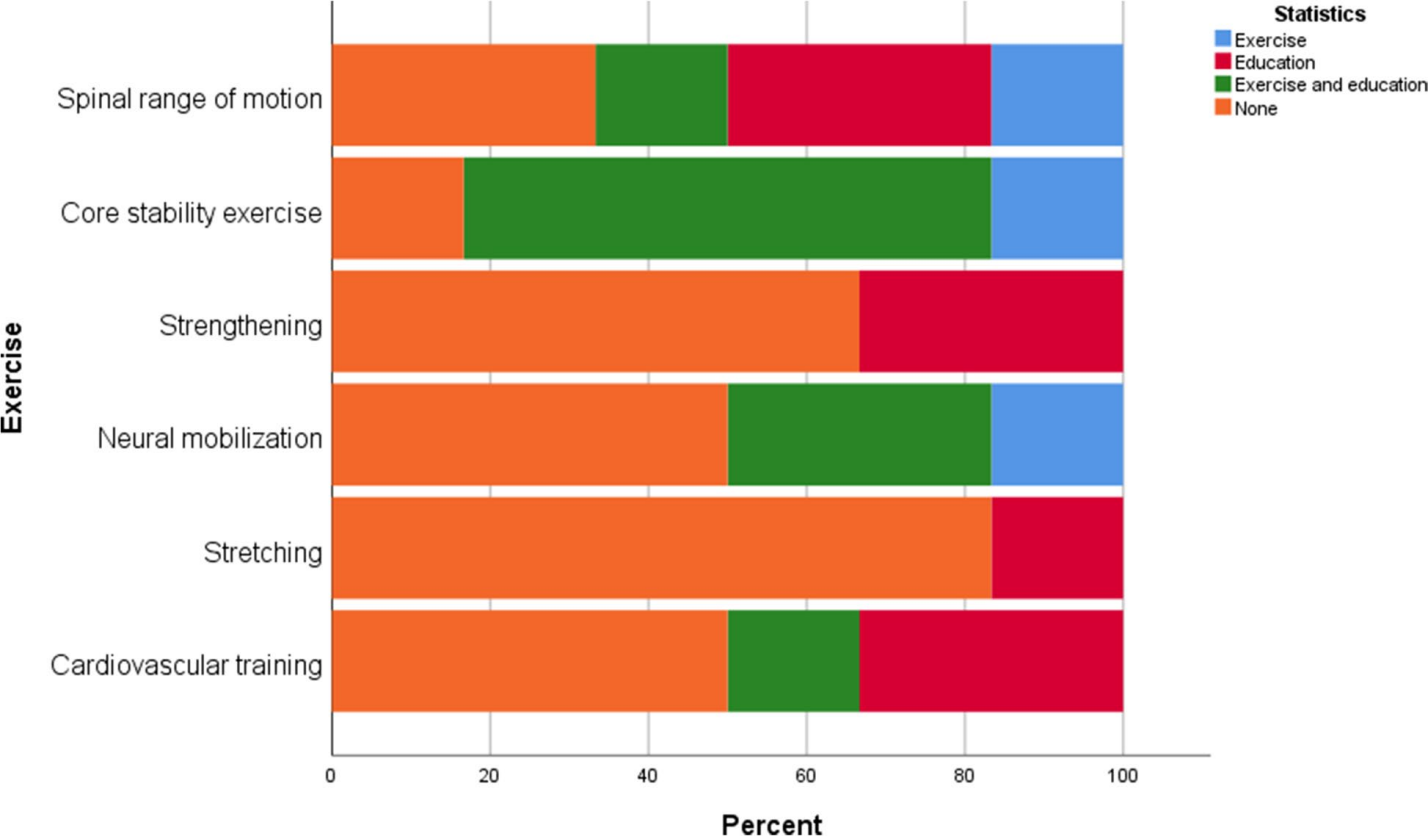
Preoperative rehabilitation content

**Fig. 4:**
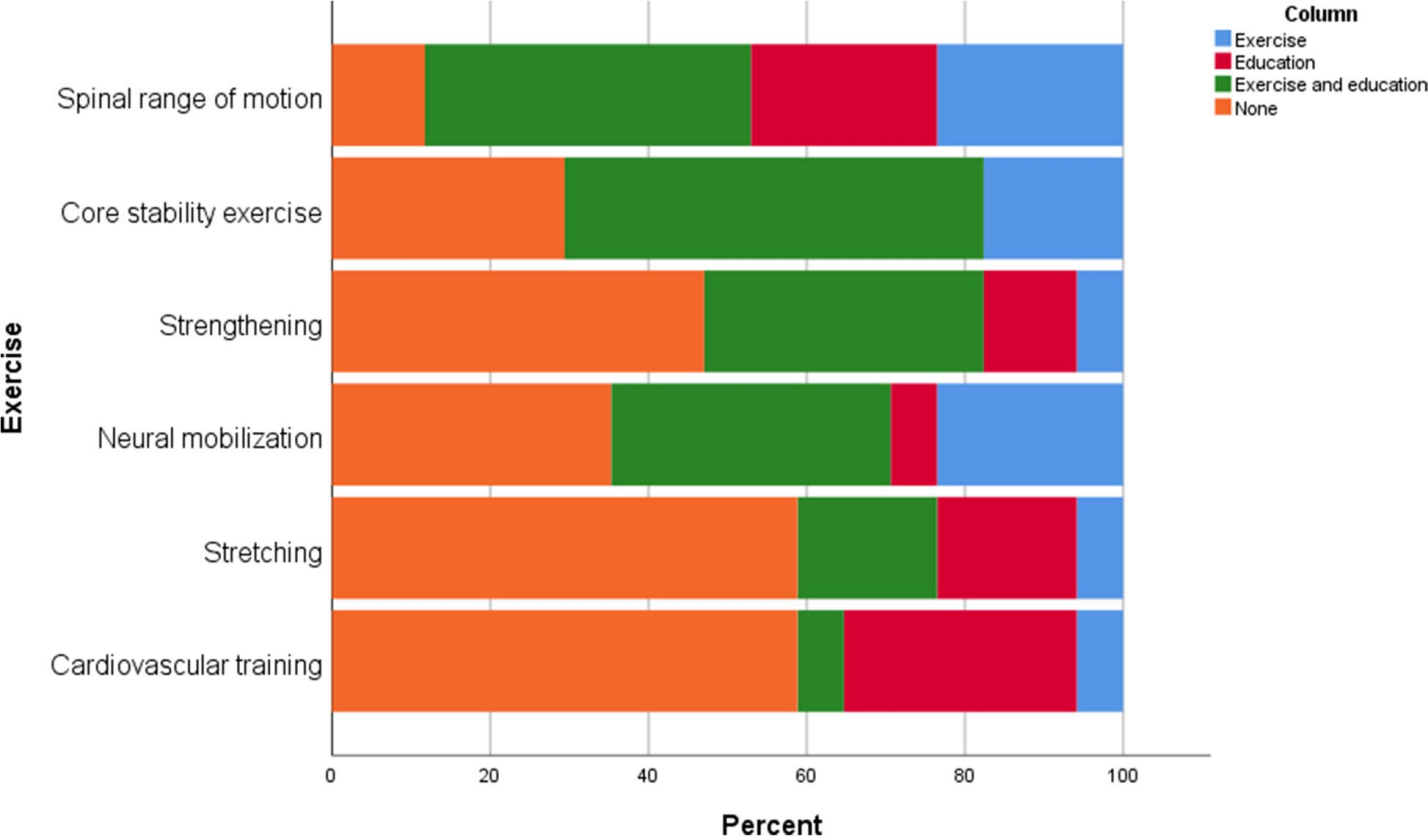
Postoperative physiotherapy content (inpatients)

**Fig. 5:**
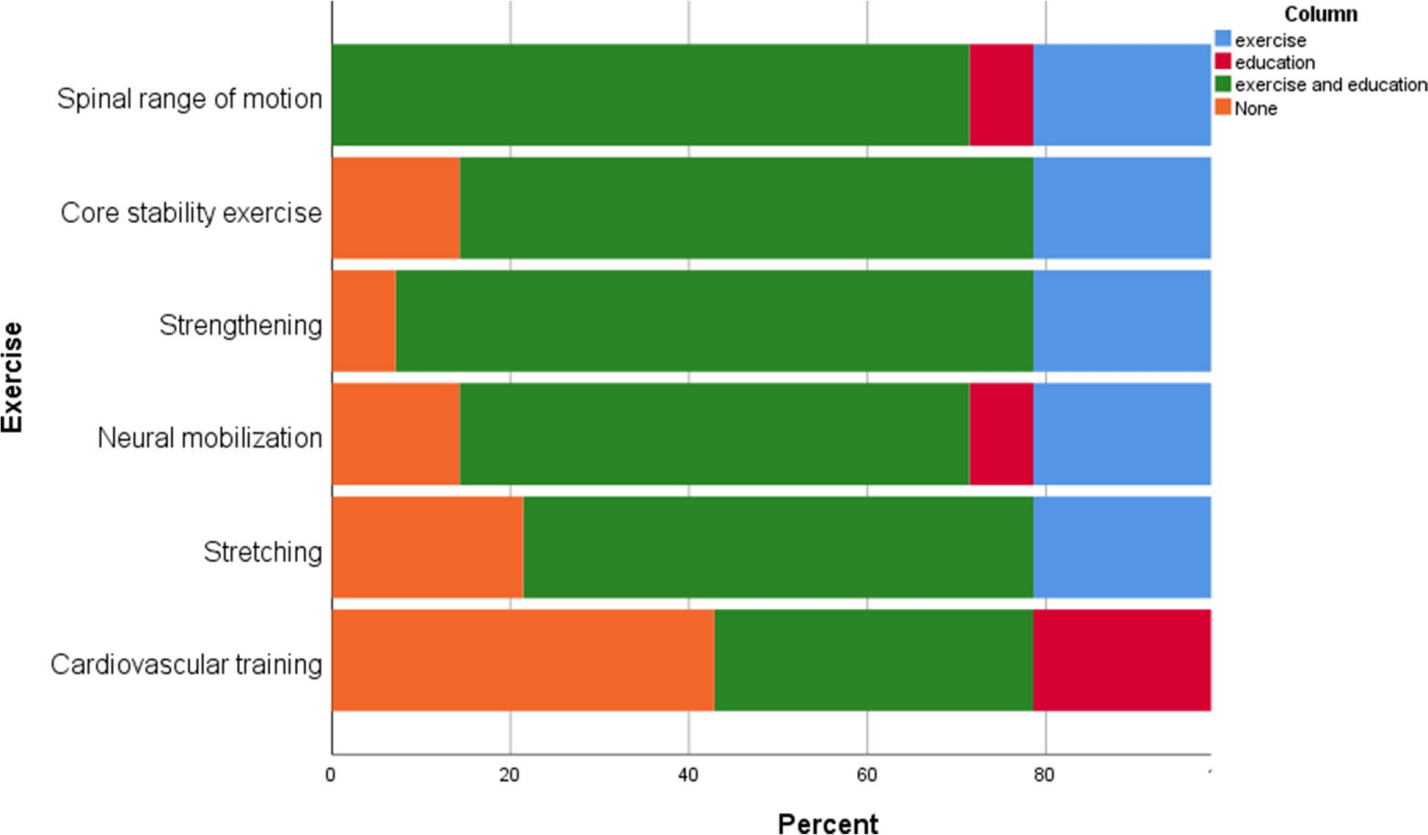
Postoperative physiotherapy content (outpatients)

All centers provided education and exercises reflecting the APTA guidelines, except one that offered only education (Table 1). Preoperative rehabilitation focused mainly on education (Fig. 3), while Postoperative inpatient rehabilitation focused on both exercise and education (Fig. 4), and postoperative outpatient rehabilitation focused more on exercises (Fig. 5). The most common preoperative and postoperative inpatient exercises were core stability and spinal range of motion (Figs. 3 and 4), whereas the spinal range of motion and strengthening as a postoperative outpatient (Fig. 5).

### Mobility and functional task

Advice about mobility and functional tasks was provided during both preoperative and postoperative inpatient rehabilitation. All centers that provided preoperative and postoperative inpatient rehabilitation provided education in bed mobility, chair mobility, ambulation, and stairs.

### Advice about restrictions

Advice regarding postoperative restrictions was similar across centers providing preoperative and postoperative inpatient rehabilitation. Advice included lifting, sitting, returning to work, resuming usual activities, and driving (Table 2). Advice about restrictions provided in the outpatient sessions varied between centers (Table 2).

**Table 2:** Rehabilitation advice provided about postoperative restrictions on activities of daily living following lumbar discectomy

	Rehabilitation timing		
Advice about restrictions	Preoperative N/6 (%)	Postoperative (inpatients) N/17 (%)	Postoperative (outpatients) N/14 (%)
**Back movements**	5 (83%)	15 (88%)	8 (57%)
**Lifting**	6 (100%)	17 (100%)	11 (79%)
**Sitting**	6 (100%)	16 (94%)	9 (64%)
**Walking**	5 (83%)	15 (88%)	6 (43%)
**Return to work**	6 (100%)	16 (94%)	10 (71%)
**Resuming usual activity**	6 (100%)	16 (94%)	12 (86%)
**Resuming driving**	6 (100%)	16 (94%)	12 (86%)
**Resuming sex**	3 (50%)	9 (53%)	7 (50%)

## Discussion

The aim of this study was to determine the provision and type of rehabilitation for patients undergoing lumbar discectomy in UK neurosurgical centers. The main findings of this survey were that all neurosurgery centers that contributed provided some sort of postoperative inpatient rehabilitation for patients undergoing LD surgery. In contrast, one in three provided preoperative rehabilitation, and more than half provided postoperative outpatient rehabilitation. Seven centers (41%) followed an ICP. The content varied by service in terms of the type of exercise recommended, information provision, and advice on restrictions post-therapy.

The strengths of our survey were that it was the first to evaluate preoperative rehabilitation following LD in the UK. Our questions reflect that of Gilmore et al. (2016) so a comparison between the UK and Australia can be made. Private and NHS centers have been included, providing a more reflective picture of practice.

The limitations of our survey include response rate. Some researchers consider an acceptable response rate of between 60 to 75% [19, 20]. On average, online survey response rates are 11% below mail and phone surveys, and rates as low as 2% have been reported [21]. The total population of neurosurgery centers in the UK was unclear making the response rate difficult to calculate. However, most of the survey findings were in line with the literature [11, 12], which contributes perhaps to confidence about the data quality. Efforts made to increase the response rate included: telephoning, emailing, and using social media. It is possible that those centers that did not reply may have had a different rehabilitation timing.

Information about rehabilitation and its timing was obtained by self-report and could have been subject to potential recall errors. Additionally, different teams may be responsible for preoperative and postoperative rehabilitation in each center; it is difficult to determine whether all teams were involved in responding. Finally, whilst most regions of England and Wales were represented, there may be differences between centers in terms of population, number, and type of surgery; caution is needed when generalizing the findings to the whole of the UK.

In comparison to previous surveys, our findings showed that 9 (53%) of centers provided routine postoperative outpatient rehabilitation, compared to (44%) in a UK survey undertaken 14 years ago [11]; similar also to the proportion in a recent Australian survey (49%) [12]. We found that one in three provided routine preoperative rehabilitation. There are no previous UK data to our knowledge, though the proportion is similar to that observed in the Australian survey (39%) [12]. Postoperative inpatient rehabilitation was provided in all centers, similar to previous findings in the UK [11] and Australia [12].

Similarly, in the current survey, of the six UK centers that provided a preoperative service, about half invited all patients, others based decisions about invitation on a combination of factors external to the patient, including staffing, time limitation, and staff experience, and factors internal to the patient, including functional status, mobility, and distance to travel. Patients living far away from neurosurgery centers are not always invited or can travel to the appointment, and it is not clear whether they have access to local services. Although there are programs helping to improve access to specialist rehabilitation services, such as the UK Rehabilitation Outcomes Collaborative (UKROC), patients in some areas cannot access the right rehabilitation services [22].

In the Australian survey, Gilmore et al. (2016) reported that preoperative rehabilitation was provided at 39% (25/64) of hospitals either in a preadmission clinic (46%, 12/25) or following admission to hospital (54%, 14/25) and that the most common reason for not providing preoperative rehabilitation was a lack of opportunity due to the patient preadmission or admission process [12]. We also found that some neurosurgical centers did not provide rehabilitation for the same reason. Some centers reported that if a patient was admitted over the weekend or as an emergency case, they would not have the opportunity to receive preoperative or postoperative inpatient rehabilitation. Weekend surgery and the lack of a corresponding seven-day rehabilitation service in some centers may be one reason for the variation in treatment provision.

Preoperative rehabilitation has been shown to be effective in other settings, including intra-abdominal surgery [23–25]; cardiac surgery [26]; cancer surgery [27, 28]; hip and knee total arthroplasty 29, 30; and anterior cruciate ligament reconstruction surgery [31]. To date, however, there are limited data concerning the impact of therapy on outcomes for patients with LD, for which further research is needed.

Evidence showed that preoperative rehabilitation is provided for other back surgery. Current practice in the UK for lumbar fusion surgery showed that 94% of surgeons reported that their patients were seen preoperatively [32]. Almost half the surgeons provided written information sheets/booklets for patients preoperatively [32]. This was comparable to Dutch and Swedish practice, where most surgeons provided preoperative information on postoperative mobilization, and almost one-third recommended preoperative rehabilitation, one third referred rarely/sometimes, and one third did not recommend it [33]. There is no similar study to explore the surgeon’s opinion about LD in the UK.

In our survey, just under half of the centers followed ICPs or a healthcare protocol. We did not ask about details of individual ICPs. However, there is evidence of variation in the composition and application of ICPs in spinal surgery [34]. The National Pathways Association defines ICPs as MDT practice-based ‘locally agreed, on guidelines and evidence, where available, for a specific patient/client group’ [35]. ICPs work to guide health and social care providers through an outline of planned procedures for either a group of patients or a particular process through complex systems [36]. Advantages of ICPs include greater consistency in practice, improved continuity of care, monitoring standards of care, improved documentation, implementation of evidence-based practice, improved teamwork, reduced duplication, enhanced management of risk, and delivery of care [37–39]. Presently there is not a published evidence-based ICP for LD surgery [34], which may explain variations in provision.

The APTA guidelines recommend general postoperative exercises [8]. Adherence to guidelines may improve patient care quality by ensuring the best evidence-based care is offered, reducing ineffective and/or unsafe interventions, improving health outcomes, and lowering healthcare utilization and cost [40, 41]. The current survey showed that all centers except one reflected the available guidelines in terms of postoperative rehabilitation. We found variation between centers in the type of exercises provided preoperatively and postoperatively; however, APTA guidelines indicated that the general exercises are acceptable as one type of exercise was not found to be superior to another. This variation probably reflects the lack of robust evidence which inform the APTA guidelines. The findings are similar to previous research that showed variability in individual exercises and advice prescribed regarding movement and activity restrictions [12]. This could be because clinicians responded to the clinical presentation of patients rather than following external guidelines such as APTA.

There was a difference in preoperative and postoperative rehabilitation. Education alone dominated preoperative rehabilitation, whereas exercise and education together focused more during postoperative inpatient, and exercise dominated postoperative outpatient rehabilitation. As outlined, the evidence base to inform the content of lumbar surgery rehabilitation is weak, and it remains unclear which interventions, or combination of interventions, impact the outcome. Studies have shown that including education in a rehabilitation program for lumbar surgery patients reduces anxiety, increases patient empowerment and satisfaction, and results in positive outcomes [42–44]. APTA Guidelines refer to education as ‘postoperative precautions, exercise, and resuming physical activity’ [8]. In the current survey we did not define the term education. We asked the centers if they provided education and followed this by asking about its timing, format, and whether it included exercise or restrictions; the term education reflected the description in the APTA guidelines.

Increasing patient knowledge is an important component of management [45]. In our survey, education was provided in the form of written handouts or verbal information during face-to-face sessions. Consistent with previous studies, handouts were the most frequent method of delivery [11, 12, 46]. Handouts can improve patients’ knowledge, satisfaction, and treatment adherence [47] though the quality of written patient information given to NHS patients ahead of and after lumbar spine surgery has been criticized [48, 49]. Suggestions have been made to improve the material, including recommendations for activity advice following LD particularly [49]. An evidence based handout developed by an expert group has been recently assessed for efficacy in a pilot RCT [50, 51].

Although described as rehabilitation, we found that preoperative rehabilitation tends to focus on providing information and preparing the patients for postoperative events. This reflects the fact that the majority of patients who have been listed for surgery have already received rehabilitation as part of conservative management of their back pain. Therefore, preoperative rehabilitation tends to focus on education for the postoperative period, including mobility and functional training. There is also no evidence to suggest how the content of the preoperative education should be provided.

As expected, all centers reported providing advice about postoperative restrictions. Advice provided preoperatively and as an inpatient, postoperatively, was generally broad; however, advice during outpatient rehabilitation was less consistent and possibly more specific and targeted to individual patient needs. A lack of information about what to expect during postoperative recovery has been linked with patient anxiety and depression symptoms both before and after surgery [45]. This is relevant because preoperative anxiety and depression are linked to higher pain and physical impairments and lower health-related quality of life in spinal surgery patients [52, 53]. Evaluations of the impact of patient information have shown that they improve patient knowledge and satisfaction [54–56], and in acute conditions, they enhance adherence to treatment [57, 58].

This study provides an insight into current practice. More evidence is needed to provide clearer and more detailed guidelines, including those relevant to the UK. Future research should aim to identify and evaluate the optimal timing, content, frequency, and format of LD surgery rehabilitation. Barriers and facilitators to guideline implementation would also facilitate standardization of care.

## Conclusions

This study has provided descriptive data on the current rehabilitation for patients undergoing LD in the UK. The survey findings highlight inequity in the provision and add to the global picture of current practice. All neurosurgical centers in this survey provided rehabilitation for patients with LD surgery with therapy primarily delivered postoperatively. One in three centers provided preoperative rehabilitation. Preoperative therapy tended to include more general advice, whereas this was probably more targeted to patient needs in the postoperative setting. There was evidence of variability in the type of exercises prescribed in different centers. Further research is needed to determine the optimum timing, content, frequency and format and of rehabilitation for patients undergoing LD surgery.

## Supplementary Information

**Additional file 1.** Survey questionnaire.

## Data Availability

The datasets generated during and/or analyzed during the current study are available from the corresponding author on reasonable request.
