# Supplementary material for: Physical therapy of patients undergoing first-time lumbar discectomy: a survey of current UK practice
Source: BMC Musculoskelet Disord. 2022 May 27;23:503. doi: 10.1186/s12891-022-05346-1 (PMC9137089; doi:10.1186/s12891-022-05346-1)
Supplement: Supplementary file 1 — Additional file 1. Survey questionnaire. [file 12891_2022_5346_MOESM1_ESM.docx]

| Discectomy survey questions |
| --- |
| 1. If you would like to complete the survey, please select the agree option below.  - Agree - Disagree |
| Section 1: Demographics |
| 1. What is the centre's name where the patients you treat had their lumbar discectomy surgery (e.g., name of the acute hospital)?   --------------------------------------------------------------------------------------- |
| 1. On average, how many patients had lumbar surgery at this centre per week?   Less than 1 1-6 6-10 11-15 20 or greater Don’t know  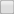Lumbar  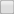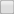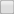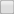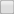microdiscectomy  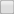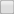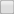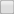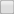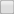Lumbar  discectomy  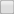  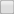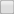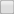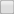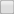Laminectomy  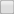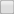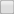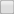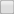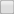Spinal fusion |
| 1. Who performs lumbar discectomy surgery in your hospital? (Tick all that apply)   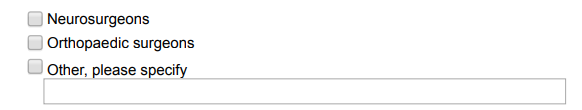 |
| 1. Who makes decisions about referrals to physiotherapy, either before or after lumbar discectomy surgery? (Tick all that apply)   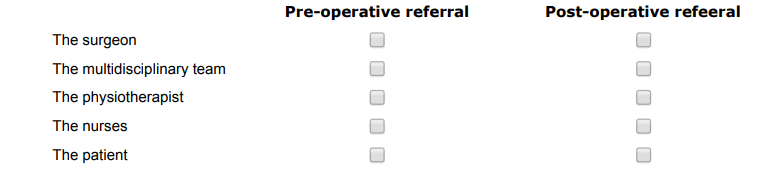 |
| 1. Do you follow an integrated care pathway or health care protocol that includes physiotherapy for lumbar discectomy surgery patients?  - Yes - No - Unsure |
| The following questions relate to patients who are seen, 1) preoperatively, 2) postoperatively as an inpatient, and 3) postoperatively as an outpatient. You will be asked to indicate whether your patients are seen during these times. Depending on your response, you will be asked to answer a series of questions related to physiotherapy during those times. |
| 1. When does a physiotherapist see discectomy patients? (Tick all that apply)  - Preoperatively as an outpatient or preoperatively as an inpatient (participants will be taken to section 2A or 2B) - Postoperatively as an inpatient, i.e., before discharge (participants will be taken to section 3A or 3B) - Postoperatively as an outpatient, i.e., following discharge (participants will be taken to section 4A or 4B) - None of the above (participant’s will be taken to section 5) |
| Section 2: Physiotherapy Intervention (Preoperative out or inpatient rehabilitation) |
| 1. How many lumbar discectomy patients are seen by a physiotherapist preoperatively?  - All (participants will be taken to section 2A) - Some (participants will be taken section 2B) |
| Section 2A: Physiotherapy Intervention (Preoperative out or inpatient rehabilitation) |
| 1. What are the goals of preoperative physiotherapy? (Tick all that apply)  - To demonstrate the exercises prescribed in the postoperative setting - To aid in preoperative pain management - To achieve preoperative ROM, stability, or strength gains - To maximize preoperative function - Other, please specify   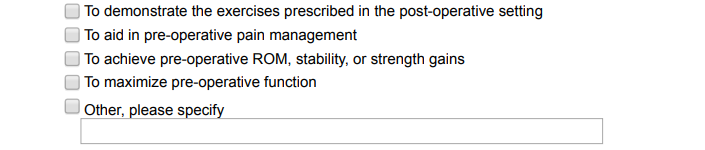 |
| 1. When are lumbar discectomy patients seen for physiotherapy, preoperatively? (Tick all that apply)   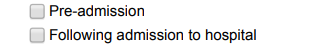 |
| 1. What format does the physiotherapy take preoperatively?  - One-to-one - Class |
| 1. How many times are lumbar discectomy patients usually seen for physiotherapy, preoperatively?  - Once - Twice - Three or more times |
| 1. Does preoperative physiotherapy include any education? (Tick all that apply)   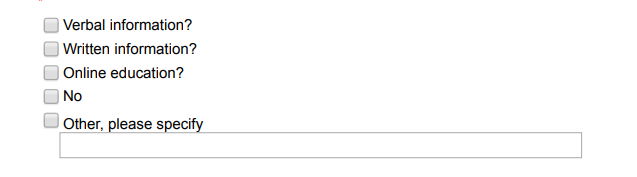 |
| 1. 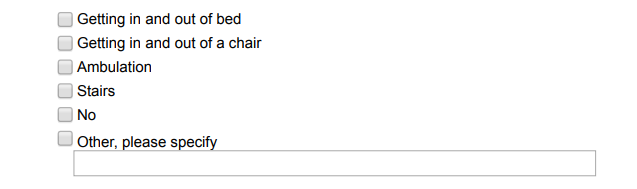Does preoperative physiotherapy include mobility and functional tasks? please indicate what areas are covered. (Tick all that apply) |
| 1. 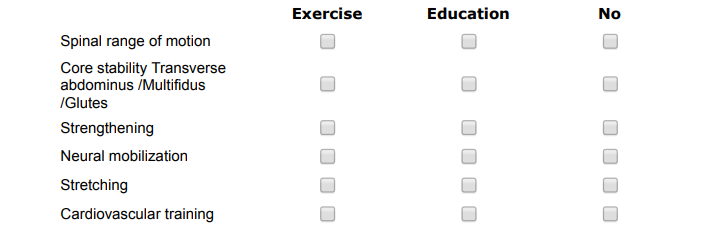Does the preoperative physiotherapy include: (Tick all that apply) |
| 1. Do you advise patients during preoperative physiotherapy about the likely restrictions in their activity that they will be required to follow once they have had their surgery?   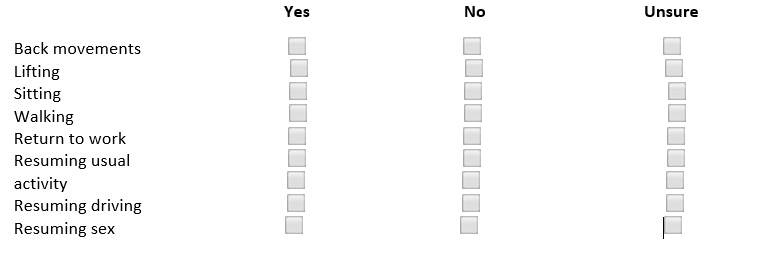 |
| Section 2B: Physiotherapy Intervention (Preoperative out or inpatient rehabilitation) |
| 1. Please indicate what external factors influence the decision to see lumbar discectomy patients by a physiotherapist preoperatively. (Tick all that apply)   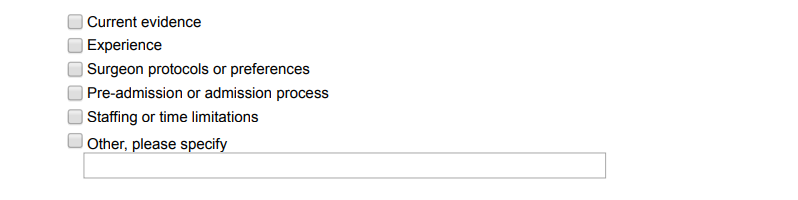 |
| 1. In terms of patient-related factors: Which of the following criteria are used to identify lumbar discectomy patients requiring physiotherapy preoperatively?   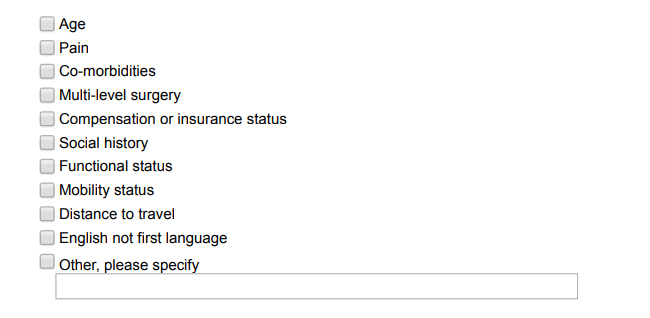 |
| 1. What are the goals of preoperative physiotherapy? (Tick all that apply)  - To demonstrate the exercises prescribed in the postoperative setting - To aid in preoperative pain management - To achieve preoperative ROM, stability, or strength gains - To maximize preoperative function - Other, please specify   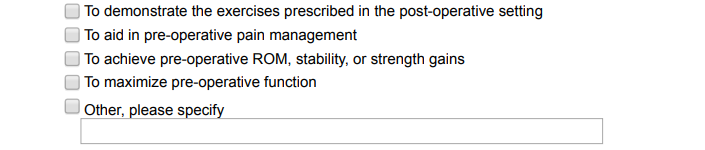 |
| 1. When are lumbar discectomy patients seen for physiotherapy preoperatively? (Tick all that apply)   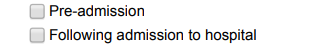 |
| 1. What format does the physiotherapy take preoperatively?  - One-to-one - Class |
| 1. How many times are lumbar discectomy patients usually seen for physiotherapy preoperatively?  - Once - Twice - Three or more times |
| 1. Does preoperative physiotherapy include any education? (Tick all that apply)   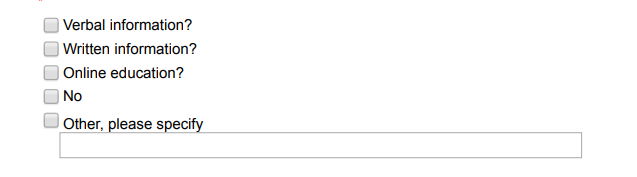 |
| 1. 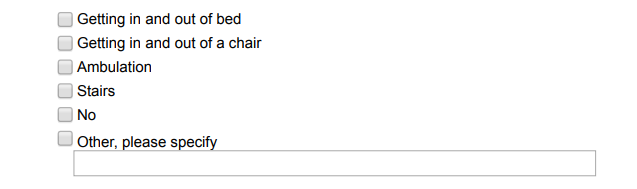Does preoperative physiotherapy include mobility and functional tasks? please indicate what areas are covered. (Tick all that apply) |
| 1. 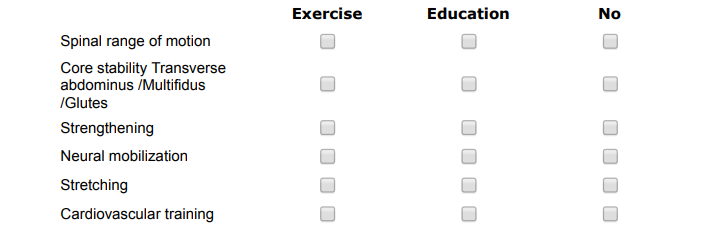Does the preoperative physiotherapy include: (Tick all that apply) |
| 1. Do you advise patients during preoperative physiotherapy about the likely restrictions in their activity that they will be required to follow once they have had their surgery?   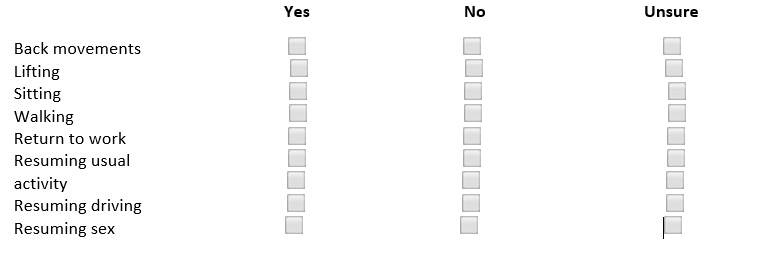 |
| Section 3: Physiotherapy Intervention (Postoperative inpatients, immediately post  operatively in the acute setting i.e. before discharge) |
| 1. How many first-time lumbar discectomy patients are seen by a physiotherapist immediately postoperatively during their inpatient stay?  - All (participants will be taken to section 3A) - Some (participants will be taken section 3B) |
| Section 3A: Physiotherapy Intervention (Postoperative inpatients, immediately post  operatively in the acute setting i.e., before discharge) |
| 1. What are the goals of physiotherapy immediately postoperatively? (Tick all that apply)  - To demonstrate the exercises prescribed in the postoperative setting - To aid in preoperative pain management - To achieve preoperative ROM, stability, or strength gains - To maximize preoperative function - To ensure the patient is safe to be discharged home - Other, please specify   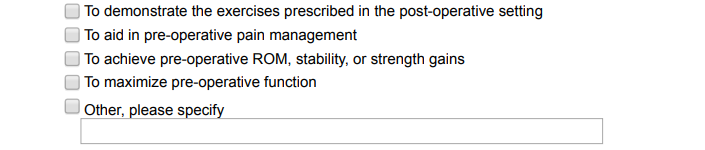 |
| 1. How soon after surgery are patients first seen by a physiotherapist?  - Day of surgery - Day 1 - Day 2 - Day 3 or later |
| 1. How many times per day are patients usually seen postoperatively?  - Once - Twice - Three or more times - Other, please specify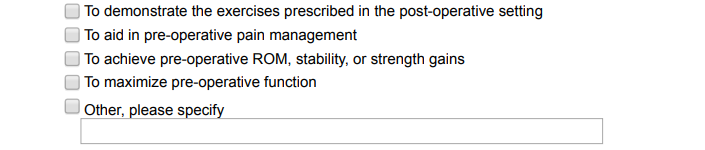 |
| 1. Does the postoperative physiotherapy include any education? (Tick all that apply)  - Verbal information? - Written information? - Online information? - No - Other, please specify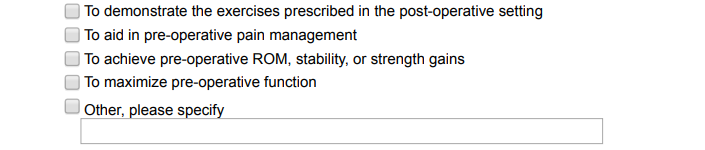 |
| 1. Does the postoperative physiotherapy include mobility and functional tasks? please indicate what areas are covered. (Tick all that apply)  - Getting in and out of bed - Getting in and out of a chair - Ambulation - Stairs - No - Other, please specify   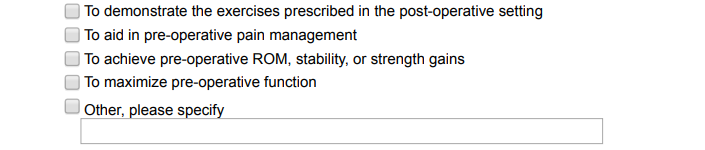 |
| 1. Does the postoperatively physiotherapy include: (Tick all that apply)   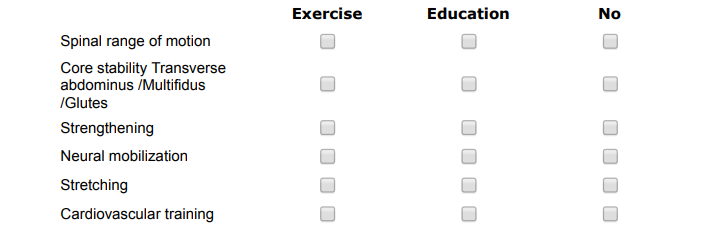 |
| 1. Do you advise patients during postoperative physiotherapy about the likely restrictions in their activity that they will be required to follow once they have had their surgery?   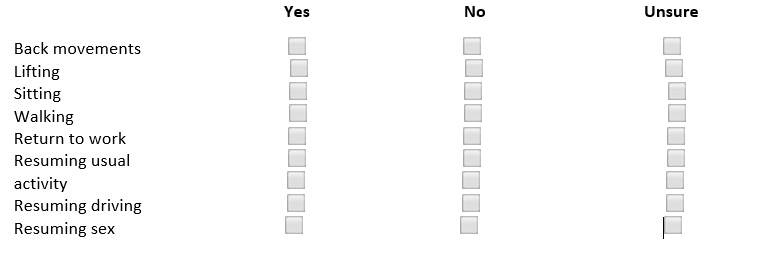 |
| Section 3B: Physiotherapy Intervention (Postoperative inpatients, immediately postoperatively in the acute setting i.e., before discharge) |
|  |
| 1. Please indicate what external factors influence the decision to see first time lumbar discectomy patients by a physiotherapist immediately postoperatively. (Tick all that apply)  - Current evidence - Experience - Surgeon protocols or preferences - Pre-admission or admission process - Staffing or time limitations - 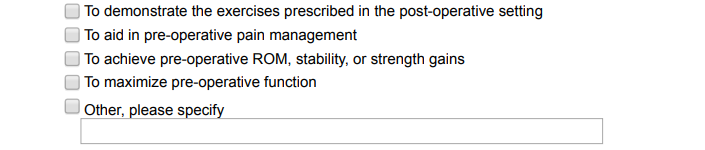Other, please specify |
| 1. In term of patient related factors: Which of the following criteria are used to identify first time lumbar discectomy patients that require physiotherapy immediately postoperatively? (Tick all that apply)  - Age - Pain - Comorbidities - Multi-level surgery - Compensation or insurance status - Social history - Functional status - Mobility status - Other, please specify   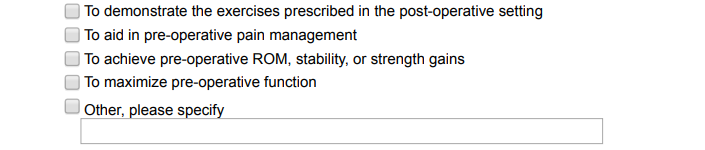 |
| 1. What are the goals of physiotherapy immediately postoperatively? (Tick all that apply)  - To demonstrate the exercises prescribed in the postoperative setting - To aid in preoperative pain management - To achieve preoperative ROM, stability, or strength gains - To maximize preoperative function - To ensure the patient is safe to be discharged home - Other, please specify   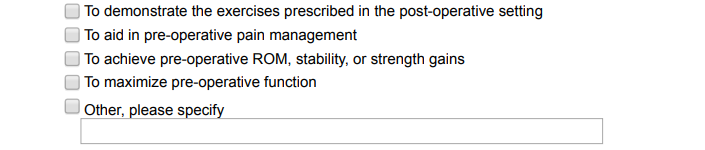 |
| 1. How soon after surgery are patients first seen by a physiotherapist?  - Day of surgery - Day 1 - Day 2 - Day 3 or later |
| 1. How many times per day are patients usually seen postoperatively?  - Once - Twice - Three or more times - Other, please specify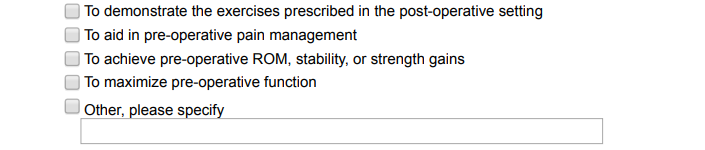 |
| 1. Does the postoperative physiotherapy include any education? (Tick all that apply)  - Verbal information? - Written information? - Online information? - No - Other, please specify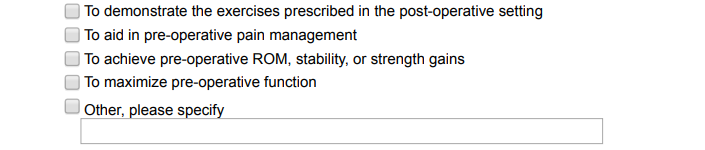 |
| 1. Does the postoperative physiotherapy include mobility and functional tasks? If so, please indicate what areas are covered. (Tick all that apply)  - Getting in and out of bed - Getting in and out of a chair - Ambulation - Stairs - No - Other, please specify   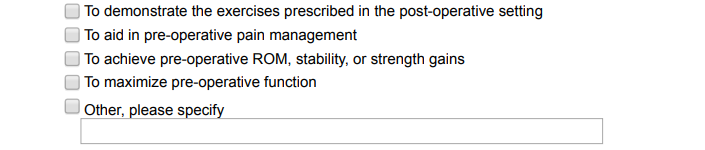 |
| 1. Does the postoperatively physiotherapy include: (Tick all that apply; Please tick if the physiotherapy is delivered as an exercise session, an education session or both).   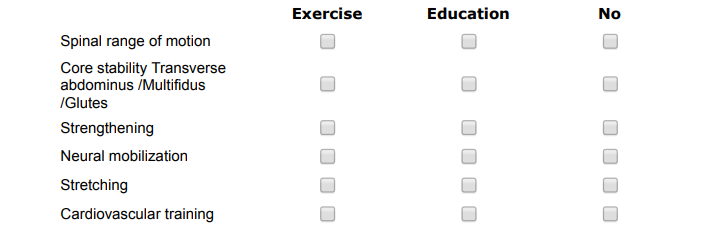 |
| 1. Do you advise patients during postoperative physiotherapy about the likely restrictions in their activity that they will be required to follow once they have had their surgery?   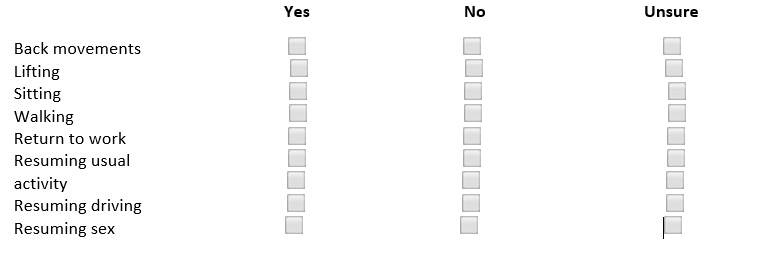 |
| Section 4: Physiotherapy Intervention (Postoperative outpatient rehabilitation i.e., following  discharge) |
| 1. How many first-time lumbar discectomy patients are seen by an outpatient physiotherapist?  - All (participants will be taken to section 4A) - Some (participants will be taken section 4B) |

| Section 4A: Physiotherapy Intervention (Postoperative outpatient rehabilitation i.e., following  discharge) |
| --- |
| 1. What are the goals of commencing outpatient physiotherapy? (Tick all that apply)  - To start postoperative exercises that were prescribed preoperatively - To start postoperative exercises that were prescribed immediately postoperatively. - To aid in postoperative pain management - To achieve postoperative ROM, stability, or strength gains - To maximize postoperative function - Other, please specify   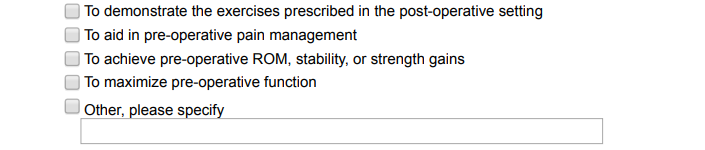 |
| 1. When does outpatient physiotherapy commence?  - Within 2 weeks following discharge - 2 – 6 weeks following discharge - 7 weeks or greater - Variable dependent on patient need - Other, please specify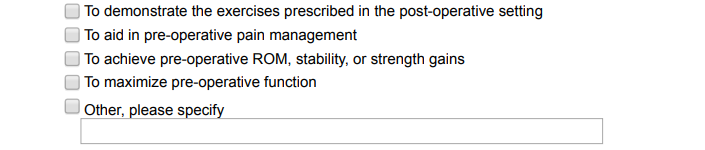 |
| 1. How are they seen in the outpatient physiotherapy?  - One-to-one - Class - Both |
| 1. How many times per week are patients routinely seen in the outpatient physiotherapy clinic?  - Once - Twice - Three or more times - less than once per week, please specify: 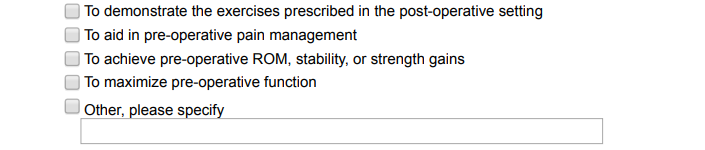 |
| 1. Does the outpatient physiotherapy include any education? (Tick all that apply)  - Verbal information? - Written information? - Online information? - No - Other, please specify   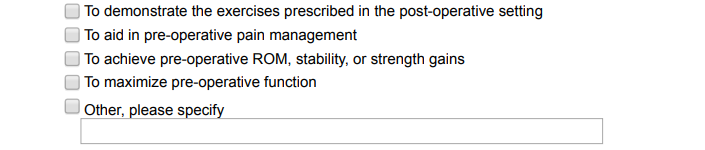 |
| 1. Does the outpatient physiotherapy include? (Tick all that apply)   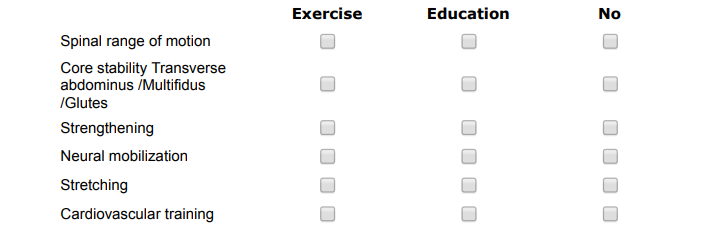 |
| 1. Are any of the following restrictions applied to your first-time lumbar discectomy patients?   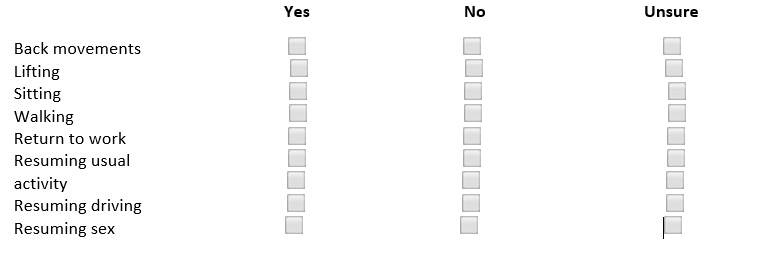 |
| Section 4B: Physiotherapy Intervention (Postoperative outpatient rehabilitation i.e., following  discharge) |
| 1. Please indicate what external factors influence the decision to see first time lumbar discectomy patients by an outpatient physiotherapist. (Tick all that apply)  - Current evidence - Experience - Surgeon protocols or preferences - Pre-admission or admission process - Staffing or time limitations - 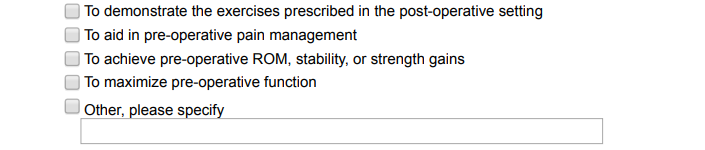Other, please specify |
| 1. In term of patient related factors: Which of the following criteria are used to identify first time lumbar discectomy patients that require outpatient physiotherapy? (Tick all that apply)  - Age - Pain - Co-morbidities - Multi-level surgery - Compensation or insurance status - Social history - Functional status - Mobility status - Other, please specify   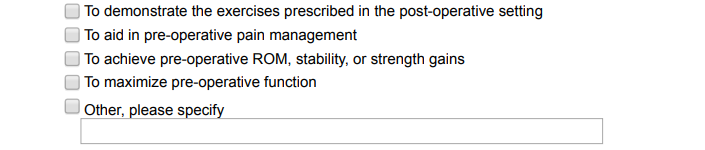 |
|  |
| 1. What are the goals of commencing outpatient physiotherapy? (Tick all that apply)  - To start postoperative exercises that were prescribed preoperatively - To start postoperative exercises that were prescribed immediately postoperatively. - To aid in postoperative pain management - To achieve postoperative ROM, stability, or strength gains - To maximize postoperative function - Other, please specify   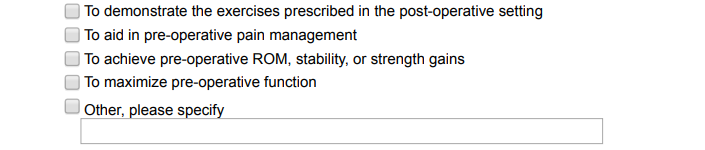 |
| 1. Approximately when are first time lumbar discectomy patients referred for outpatient physiotherapy, when does it generally commence?  - Within 2 weeks following discharge - 2 – 6 weeks following discharge - 7 weeks or greater - Variable dependent on patient need - Other, please specify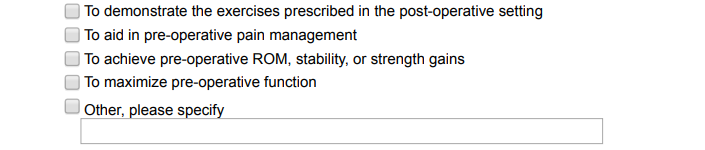 |
| 1. How are they seen in the outpatient physiotherapy?  - One-to-one - Class - Both |
| 1. How many times per week are patients routinely seen in the outpatient physiotherapy clinic?  - Once - Twice - Three or more times - less than once per week, please specify: 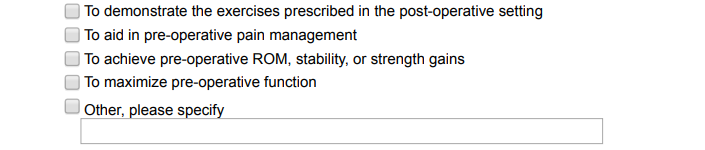 |
| 1. Does the outpatient physiotherapy include any education? (Tick all that apply)  - Verbal information - Written information - Online information - No - Other, please specify   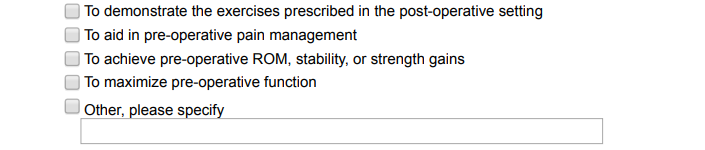 |
| 1. Does the outpatient physiotherapy include: (Tick all that apply)   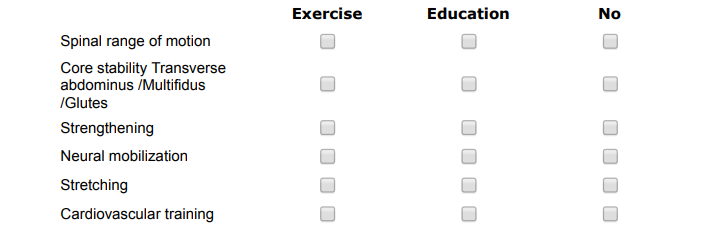 |
| 1. Are any of the following restrictions applied to your first time lumbar discectomy patients?   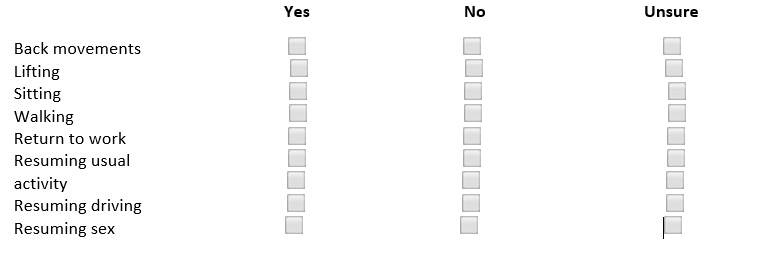 |

| Section 5: Physiotherapy Intervention is NOT Provided |
| --- |

| 1. Please indicate what factors influence the decision not to see first time lumbar discectomy patients by a physiotherapist. (Tick all that apply)  - Current evidence - Experience - Surgeon protocols or preferences - Pre-admission or admission process - Staffing or time limitations - 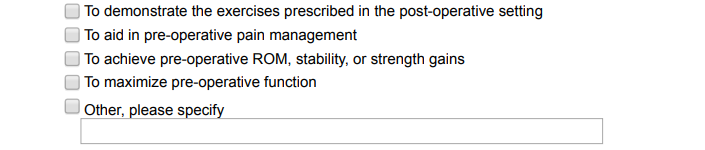Other, please specify |
| --- |
